# Peroneus Longus Versus Hamstring Tendon Graft for Anterior Cruciate Ligament Reconstruction: A Retrospective Matched Comparison

**DOI:** 10.3390/jcm14207319

**Published:** 2025-10-16

**Authors:** Mustafa Kursat Sari, Ozkan Kose

**Affiliations:** Department of Orthopedics and Traumatology, Antalya Training and Research Hospital, 07100 Antalya, Turkey; mustafakursatsari@gmail.com

**Keywords:** anterior cruciate ligament reconstruction, peroneus longus tendon, hamstring tendon, autografts, donor-site morbidity, treatment outcome, graft diameter, complication

## Abstract

**Background/Objectives**: This study aimed to compare clinical and functional outcomes of anterior cruciate ligament reconstruction (ACLR) using peroneus longus tendon (PLT) versus hamstring tendon (HT) autografts in matched cohorts. **Materials and Methods**: A retrospective matched cohort study with 1:1 nearest-neighbor matching without replacement on sex (exact), age (±3 years), and time to last follow-up (closest match) was performed on consecutive primary single-bundle ACLR cases. After exclusions and follow-up completion, 77 PLT patients were matched 1:1 with 82 HT patients based on age, sex, and follow-up. Outcomes included the International Knee Documentation Committee (IKDC) Subjective Knee Evaluation Form, Lysholm Knee Score, Tegner Activity Scale, Tampa Scale for Kinesiophobia, knee range of motion, manual strength testing, stability tests, limb circumferences, and the Single-Leg Hop test. Additionally, ankle-specific function in the PLT group was assessed using the AOFAS and FADI scores. **Results**: Graft diameter was larger with PLT (8.5 ± 0.6 mm) than with HT (8.1 ± 0.7 mm; *p* = 0.003). Both groups demonstrated significant improvement from the preoperative to the postoperative period on the IKDC, Lysholm, and Tegner scores (all *p* < 0.001). Between groups, postoperative IKDC and Lysholm scores were comparable (both *p* > 0.05), and Tegner scores did not differ significantly (*p* > 0.05). Knee flexion loss was less frequent (9.1% vs. 68.3%; *p* = 0.001), and knee flexion strength was higher (4.7 ± 0.5 vs. 4.0 ± 0.8; *p* = 0.001) in PLT. Stability tests, LSI, extension strength, and limb circumferences were similar (all *p* > 0.05). Saphenous nerve symptoms were less frequent with PLT (13.0% vs. 29.3%; *p* = 0.010). Ankle function in PLT was preserved (AOFAS 96.9 ± 7.9; FADI 97.4 ± 8.5) with mild eversion limitation in 3 patients (3.9%). Re-rupture rates were low and did not differ significantly between groups (*p* > 0.05). Infections occurred only after HT, yielding a between-group difference (*p* = 0.030). **Conclusions**: PLT autografts provide clinical and functional outcomes comparable to those of HT, with advantages including a larger graft diameter, better preservation of knee flexion function, fewer symptoms related to the saphenous nerve, and excellent ankle outcomes. PLT is a safe and effective alternative for primary ACLR.

## 1. Introduction

Anterior cruciate ligament (ACL) injuries are common in active populations and often compromise knee stability and function, making reconstruction the preferred management for patients with symptomatic instability [1]. Graft selection remains a key determinant of postoperative biomechanics, rehabilitation course, and donor-site morbidity. Autografts are generally favored over allografts in young and active patients due to superior incorporation and lower failure risk [2,3]. Among autografts, hamstring tendon (HT) constructs have been widely adopted because they avoid anterior knee pain associated with bone-patellar tendon-bone harvest and permit versatile fixation options [2,3,4].

Nevertheless, HT harvest may entail hamstring strength deficits, iatrogenic saphenous nerve injury, altered neuromuscular control, and, in some series, delayed biologic maturation of the graft [5,6,7,8]. Interest has therefore grown in alternative autografts such as the quadriceps tendon and the PLT [1,9,10]. The PLT has emerged as a promising option due to its favorable diameter, adequate tensile properties, and straightforward harvest through a small incision, while potentially preserving hamstring function for sports that require sprinting or explosive deceleration [1,5,11,12].

Despite these advantages, important questions persist regarding the safety of the PLT donor site [13,14,15]. The peroneus longus contributes to ankle eversion and plantarflexion; theoretical concerns include eversion weakness, subtle gait alterations, or balance impairments, particularly in sports that involve cutting and pivoting [16,17]. Early clinical series generally report low ankle morbidity; however, many are limited by small sample sizes, short follow-up periods, heterogeneous rehabilitation protocols, or non-standardized outcome measures [1,18,19]. Comparative studies versus HT have yielded encouraging yet inconclusive results, largely due to inconsistent adjustment for confounding, inadequate rigorous matching, and, in several instances, insufficient power for evaluating functional endpoints, comprehensive ankle-specific assessments, and reoperation rates [20,21,22,23,24,25,26].

Given these gaps, higher-quality comparisons using a rigorous methodology are needed to clarify whether PLT can match or exceed HT performance while maintaining acceptable donor-site profiles. The present retrospective cohort study was designed to compare clinical and functional outcomes after ACL reconstruction using HT versus PLT autografts in a sufficiently powered sample. It was hypothesized that PLT would be non-inferior to HT for primary functional outcomes and would not be associated with clinically meaningful decrements in ankle-related donor-site measures or other complications.

## 2. Materials and Methods

### 2.1. Patients and Study Design

This retrospective cohort study included consecutive patients who underwent primary ACLR using either a PLT or HT autograft between 2014 and 2024 at our institution. Patients were excluded if they had any prior surgery on the index or contralateral knee, such as fracture fixation; concomitant major knee pathology or procedures at the index operation that could confound outcomes including multi-ligament injuries or high tibial osteotomy; incomplete medical records or missing key outcome data; or if they received graft types other than HT or PLT autografts, including allografts, quadriceps tendon, iliotibial band, or hybrid constructs.

This study received approval from the Institutional Review Board of the authors’ institution (Approval Date and Issue: 21 November 2024; No. 8-25). All procedures conformed to the principles of the Declaration of Helsinki. Informed consent was obtained from all participants.

### 2.2. Sample Size Calculation and Cohort Assembly

A strict matching strategy and a priori sample-size calculation based on validated knee function scores were employed to minimize selection bias and optimize comparability. The a priori calculation used the International Knee Documentation Committee Subjective Knee Form (IKDC) outcomes reported by Rhatomy et al. [22] as the reference. In that study, mean (±SD) IKDC scores were 88.8 ± 9.7 in the hamstring tendon (HT) group and 92.5 ± 6.2 in the peroneus longus tendon (PLT) group, a between-group difference of 3.7 points. Using these summary statistics, the pooled standard deviation was 8.14, and the corresponding standardized effect size (Cohen’s d) was 0.45. For a two-sided comparison of two independent groups with α = 0.05, power = 0.80, and 1:1 allocation, the required sample size was 76 participants per group (total *n* = 152). Accounting for approximately 10% attrition, the target enrollment was set at 83 per group (total *n* = 166).

**Figure 1:**
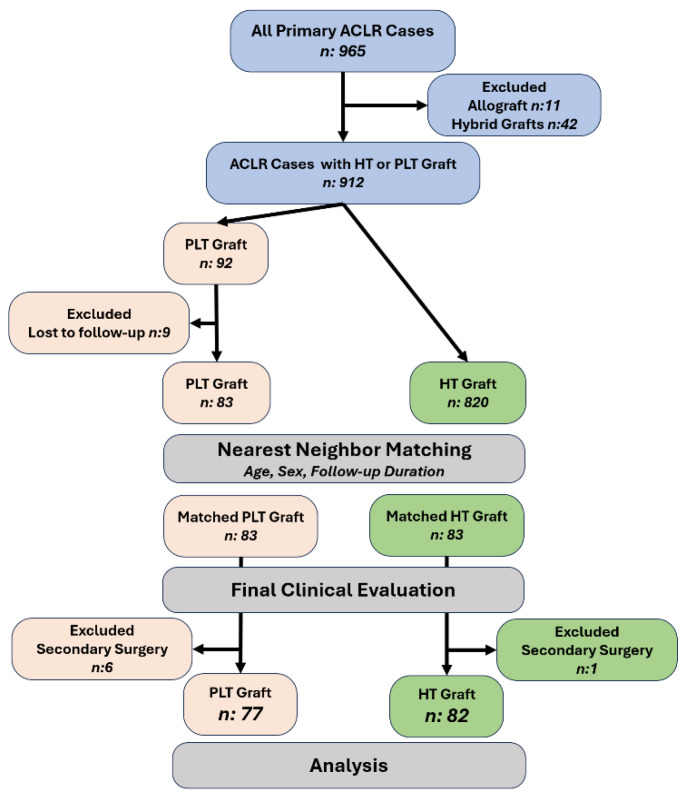
Cohort assembly and matching. Of 965 primary ACLR cases, 53 were excluded (11 allograft, 42 hybrid), leaving 912 HT or PLT autografts. After loss to follow-up (PLT *n* = 9), 83 PLT and 820 HT remained eligible. 1:1 nearest-neighbor matching (age ± 3 years, sex, follow-up duration) produced 83 PLT and 83 HT for clinical evaluation. Cases with secondary surgery were excluded (PLT, *n* = 6; HT, *n* = 1), yielding the analytic sample: PLT, *n* = 77; HT, *n* = 82. Abbreviations: ACLR, anterior cruciate ligament reconstruction; HT, hamstring tendon; PLT, peroneus longus tendon.

A total of 965 consecutive primary ACL reconstructions were screened for eligibility. After excluding 11 allograft and 42 hybrid-graft procedures, 912 autograft cases using either HT or PLT remained eligible. Of these, 92 received a PLT autograft and 820 received an HT autograft. Nine PLT cases were lost to follow-up, leaving 83 PLT cases with complete clinical and follow-up data. To minimize selection bias, we performed 1:1 nearest-neighbor matching without replacement, retaining all PLT cases (*n* = 83) and selecting matched HT comparators from the 820 eligible HT cases. Matching enforced identical sex (exact match) and age within ±3 years (hard caliper used only as an eligibility constraint), while the matching distance was defined as the absolute difference in time to last follow-up; among same-sex, age-eligible candidates, the HT case with the closest follow-up duration to each PLT case was selected. After the matching, sex was perfectly balanced by design (equally distributed in groups). Age demonstrated good balance: 28.6 ± 9.3 years (PLT) vs. 27.3 ± 9.5 years (HT); absolute standardized mean difference (ASMD) = 0.14; variance ratio (VR) = 0.96; Welch’s t = 0.89, df ≈ 164, *p* = 0.374. In contrast, follow-up duration remained shorter in the PLT group, consistent with more recent adoption of PLT grafts: 18.7 ± 14.7 vs. 27.0 ± 17.6 months; ASMD = 0.51; VR = 0.70; Welch’s t = −3.30, df ≈ 159, *p* = 0.001. Following the last clinical evaluation, cases that underwent secondary surgery during follow-up were excluded from the comparative functional analysis (PLT, *n* = 6; HT, *n* = 1), yielding a final analytic sample of 77 PLT and 82 HT patients (total *n* = 159). The cohort assembly and matching workflow are shown in Figure 1**.**

### 2.3. ACLR Technique

Procedures were performed with the patient supine under general or spinal anesthesia and tourniquet control. A systematic diagnostic arthroscopy was completed before graft preparation to confirm ACL rupture. In all cases, the femoral tunnel was created through the anteromedial portal, and femoral fixation employed a suspensory cortical button. Among 77 PLT reconstructions, 69 used a fixed-loop EndoButton and 8 an adjustable-loop device; among 82 HT reconstructions, 52 used an adjustable-loop device and 30 a fixed-loop EndoButton. Tibial fixation consisted of a bioabsorbable interference screw in all patients. To augment tibial fixation, a U-staple was additionally used in 154 of 159 cases. Concomitant meniscal and chondral lesions, when present, were treated after the graft was passed through the femoral and tibial tunnels but before tibial fixation; the procedure was then completed with tibial fixation. A suction drain was placed in all patients, and a compressive bandage was applied.

### 2.4. Graft Harvesting and Preparation

PLT graft harvest was performed according to patient anatomy and surgeon preference via supramalleolar (*n* = 13), inframalleolar (*n* = 49), or combined (*n* = 15) incisions [14]. Following blunt subcutaneous dissection, the peroneus longus and brevis tendons were identified and the common sheath opened longitudinally. The peroneus longus was then released distally; in most cases, a tenodesis to the peroneus brevis was carried out in neutral ankle position using non-absorbable sutures (*n* = 55), whereas distal tenotomy without tenodesis was performed in the remainder (*n* = 22). Harvesting the PLT with tenodesis or tenotomy was entirely at the discretion of the surgeon. The graft was harvested with a tendon stripper (6/8 mm tip), irrigated, and cleared of residual muscle. Preparation included Krakow sutures at both ends and configuration as a double-strand autograft; when intraoperative diameter was insufficient (typically < 8 mm), a triple-strand construct (*n* = 9) was fashioned.

Hamstring grafts were prepared using the semitendinosus and gracilis tendons. A 3–5 cm skin incision was made 2–3 cm medial to the tibial tubercle, parallel to the flexor crease, and the pes anserinus was exposed by sharp and blunt dissection. The semitendinosus and gracilis were identified, their insertions delineated, and each tendon was secured distally with nonabsorbable whipstitch sutures. Tendons were then released proximally with a tendon stripper at the level of the myotendinous junction, taking care to preserve the sartorial fascia; a minimal fasciotomy was performed when necessary [6]. Harvested grafts were irrigated with normal saline, cleared of residual muscle, and configured to the appropriate length as a double-strand construct. At the surgeon’s discretion, both PLT and HT grafts were stored in vancomycin-soaked sponges to reduce the risk of infection in 76 of 159 cases.

### 2.5. Postoperative Rehabilitation

Immediate cryotherapy was initiated with the knee in full extension to prevent edema and hemarthrosis. The suction drain was removed on postoperative day 1–2 according to the drain output. In isolated ACLR cases, full weight-bearing was allowed as tolerated from the outset, with crutch assistance provided for comfort. From the early postoperative period, passive knee flexion and quadriceps isometric exercises were commenced, targeting full extension and up to 120° of flexion. In cases involving meniscal repair, early rehabilitation was modified: non–weight–bearing for 4–6 weeks was prescribed, and a hinged knee brace was used to permit controlled, progressive flexion while protecting the meniscal repair. During follow-up, functional assessments, clinical examination findings, and complications were monitored. Return to sports was permitted at 8–12 months, individualized based on muscle strength recovery, functional performance, and clinical stability.

### 2.6. Functional Assessments and Examination

All patients in both groups were evaluated face-to-face by the same physician using a standardized protocol. To reduce inter-rater variability, interviews, physical examination tests, measurements, and strength assessments were performed by a single orthopedic surgeon with 5 years of experience. Functional outcomes included the IKDC, Lysholm Knee Score (LKS), and Tegner Activity Scale (TAS) [27,28,29]. Kinesiophobia was assessed using the Tampa Scale of Kinesiophobia [30]. In the PLT autograft group, ankle-related function was additionally evaluated with the AOFAS score and FADI [31,32]. Thigh circumference at the level of the superior pole of the patella and maximal calf circumference were measured bilaterally to document muscle atrophy. Knee stability was assessed with the Lachman, anterior drawer, and pivot-shift tests. Laxity was graded between Grade 0 to Grade 3 (Grade 0 = negative). Knee range of motion (ROM) was measured bilaterally with a goniometer in the supine position using standard landmarks (greater trochanter–lateral femoral epicondyle–lateral malleolus). Flexion/extension loss was defined a priori as a side-to-side difference ≥5° relative to the contralateral, asymptomatic knee. Quadriceps and hamstring strength were evaluated bilaterally using manual muscle testing (MMT; Medical Research Council 0–5 scale) with examiner-applied resistance. Functional symmetry and dynamic stability were examined with the Single-Leg Hop Test on both limbs [33]. Three trials per limb were recorded after standardized familiarization; the best distance per limb was used. Limb Symmetry Index (LSI) was calculated as (involved/uninvolved) × 100. A focused neurovascular examination was performed, including a careful assessment of the sensory and motor branches of the sural, saphenous, and common peroneal nerves to screen for potential neurological complications at the donor site

### 2.7. Statistical Analysis

Continuous variables are reported as mean ± SD, categorical variables as *n* (%). Normality was assessed using the Shapiro–Wilk test and the Kolmogorov–Smirnov test. Between-group comparisons used the Student’s *t*-test for normally distributed continuous data or the Mann–Whitney U test otherwise. Categorical variables were compared using chi-square or Fisher’s exact tests (when expected counts < 5). Within-group preoperative vs. postoperative comparisons were performed using the Wilcoxon signed-rank test. For interpretability, we report the paired mean difference (Δ = postoperative − preoperative) with 95% confidence intervals (CI), calculated from the distribution of paired differences. All tests were two-sided with an α-level of 0.05. Statistical analyses were performed using IBM SPSS Statistics Base for Windows, V.23.0 (IBM Corp., Armonk, NY, USA).

## 3. Results

After matching and exclusions, 159 patients were analyzed (HT = 82; PLT = 77). Groups were comparable in terms of age, sex, side, body size, injury mechanism, ASA class, and smoking status (all *p* > 0.05). Length of stay was shorter in the PLT group (1.4 ± 1.0 vs. 3.4 ± 4.4 days; *p* = 0.001), whereas follow-up duration was longer in the HT group (27.0 ± 17.7 vs. 18.4 ± 15.0 months; *p* = 0.001) (Table 1).

Mean graft diameter was larger in PLT than in HT (8.5 ± 0.6 vs. 8.1 ± 0.7 mm; *p* = 0.004). All HT grafts were double-stranded, whereas 11.7% of PLT grafts were configured triple-stranded (*p* = 0.001). Frequencies of medial/lateral meniscal repair or meniscectomy and associated MCL grades were similar between groups (all *p* > 0.05). Operative time did not differ (93.9 ± 22.0 vs. 93.1 ± 22.8 min; *p* = 0.489). Vancomycin graft soaking was used far more frequently in the PLT cohort than in the HT cohort (55/77 [71.5%] vs. 21/82 [25.6%]; *p* = 0.0012) (Table 2). However, across the full cohort, vancomycin soaking was not associated with postoperative infection status (Fisher–Freeman–Halton exact, *p* = 0.715). When dichotomized as any infection (deep or superficial) vs. none, rates were 2.6% with vancomycin versus 6.0% without (two-sided Fisher’s exact *p* = 0.446).

Both groups showed significant within-group improvements from pre- to postoperative assessments in IKDC, Lysholm, and Tegner scores (all *p* = 0.001). Between groups, postoperative IKDC (80.6 ± 18.3 vs. 76.5 ± 19.8; *p* = 0.185) and LKS (89.5 ± 12.4 vs. 88.9 ± 12.2; *p* = 0.588) were comparable; Tegner showed a nonsignificant trend favoring PLT (5.9 ± 1.7 vs. 5.4 ± 1.7; *p* = 0.071). Knee flexion loss was less frequent in PLT (9.1% vs. 68.3%; *p* = 0.001), although the magnitude of flexion deficit among affected patients was similar (≈9° in both; *p* = 0.806). Knee flexion strength grades were higher in PLT (4.7 ± 0.5 vs. 4.0 ± 0.8; *p* = 0.001), while knee extension strength, thigh/calf circumference differences, hop test limb symmetry index, and knee stability tests (Lachman, anterior drawer, pivot shift) were not significantly different (all *p* > 0.05). Tampa kinesiophobia totals and subscores were also similar between groups (all *p* > 0.05) (Table 3).

In the PLT cohort, ankle inversion loss occurred in 3 patients (3.9%); no losses were observed for plantarflexion, dorsiflexion, or eversion. PLT-specific ankle function scores were high (AOFAS 96.9 ± 7.9; FADI 97.4 ± 8.5). Saphenous nerve symptoms were less frequent with PLT than HT (13.0% vs. 29.3%; *p* = 0.012). Rates of postoperative hemarthrosis aspiration and re-rupture were comparable (both *p* > 0.05). Infections occurred only in the HT group (superficial, 4.9%; deep, 3.7%), yielding a significant between-group difference in overall infection (*p* = 0.030) (Table 4).

## 4. Discussion

In this matched cohort study, the primary finding is that functional outcomes were comparable between groups, with both cohorts demonstrating significant improvements in IKDC, Lysholm, and Tegner scores from pre- to postoperative assessments. In accordance with the a priori hypothesis, PLT was non-inferior to HT for primary functional endpoints, while demonstrating a reduced frequency of knee flexion loss and elevated flexion strength grades. These findings are indicative of the preservation of hamstring function. The incidence of donor-site morbidity in the PLT group was low, with high ankle-specific scores (AOFAS/FADI) and only isolated, mild motion loss. This suggests that ankle safety is acceptable when contemporary harvest and tenodesis techniques are used. The complication profiles were found to be broadly similar. Infections occurred only after HT reconstructions in this series; however, vancomycin graft soaking was used substantially more often in the PLT cohort than in the HT cohort. In cohort-level analyses, vancomycin use was not significantly associated with postoperative infection, but the low event count limits precision. Accordingly, the infection signal should be interpreted cautiously, as it may reflect practice-pattern differences rather than a graft-intrinsic effect. Collectively, these results support PLT as a viable alternative autograft to HT in ACLR, offering comparable global knee function while maintaining a favorable donor-site profile.

Our findings align with the growing body of comparative evidence, indicating that PLT provides knee-specific outcomes comparable to those of HT after primary ACLR, while offering several practical advantages. Across more than ten comparative studies, postoperative IKDC, Lysholm, and Tegner scores are typically not significantly different between PLT and HT, and knee stability, as assessed by Lachman/anterior drawer/pivot-shift, is likewise comparable (Table 5) [20,21,22,23,24,25,34,35,36,37,38,39,40,41,42,43]. Many series also report larger graft diameters with PLT and better preservation of knee flexion strength, as well as less thigh atrophy, consistent with our observation of higher flexion strength and less flexion loss in the PLT cohort. Donor-site ankle morbidity with PLT is generally low, with high AOFAS/FADI scores and only isolated, transient symptoms. Collectively, these convergent results support PLT as a viable alternative to autograft, maintaining global knee function while minimizing hamstring-specific deficits. Convergent evidence from recent meta-analyses also indicates equivalence in global knee function and stability between PLT and HT after primary ACLR, along with a tendency toward greater graft diameter and reduced hamstring-related morbidity in PLT cohorts [1,18,19].

Comparative literature largely supports PLT as equivalent to HT, with potential benefits in graft size and hamstring preservation. However, a minority of studies still report results that favor HT. In a retrospective cohort study using anterior-half PLT (AH-PLT), 3-year IKDC scores were lower with PLT, despite similar laxity test results; multivariable modeling indicated that graft type, sex (smaller-stature females), and smaller tunnel/graft diameters were associated with inferior scores, underscoring the risk of undersized AH-PLT constructs in certain patients [38]. Likewise, a prospective multicenter comparison of a six-strand HT versus PLT reported higher postoperative IKDC and Lysholm scores, as well as slightly better anterior stability with HT, with comparable complication rates [43]. These differences are plausibly attributable to the greater effective graft caliber and construct stiffness in the six-strand HT configuration. Methodological heterogeneity, including follow-up duration, rehabilitation protocols, harvest/tenodesis technique, fixation constructs, and sample size/power, likely contributes to these discrepancies. Secondly, re-rupture and infection rates are uniformly low and generally similar across groups in the literature. However, these findings should be interpreted cautiously, given the potential for residual confounding and the scarcity of events.

Concerns about using the PLT as a graft largely center on the potential for post-harvest deterioration in ankle functions, donor site morbidity. The peroneal muscles (both PLT and PB) evert the hindfoot and assist in plantarflexion, and the PLT integrates these actions to help stabilize the first metatarsal head during stance [44]. Furthermore, the PLT contributes both actively and passively to “locking” the first metatarsal against the medial cuneiform [45]. Cadaveric investigations demonstrate that the PLT affects the stiffness of the medial longitudinal arch; inadequate PLT traction can compromise arch mechanics and lead to structural foot problems [46]. Consistently, transection of the PLT produces a marked increase in medial displacement of the transverse arch, or development of pes planus [47]. Moreover, PLT insufficiency has been proposed as a potential driver of severe foot dysfunction, paralleling the clinical impact seen in posterior tibial tendon failure [48]. In light of these considerations, it is theoretically plausible that harvesting the PLT, a structure integral to ankle function, could lead to postoperative functional impairment. However, in most studies using the PLT as an ACL graft, patient-reported outcome measures have not indicated clinically meaningful ankle complaints. Moreover, beyond subjective indices, several series incorporating objective assessments, such as gait analysis and isokinetic/strength testing, have likewise failed to demonstrate a significant decrement in ankle performance [14,17,41,49]. Although the PLT is not a dispensable muscle, we posit that the tenodesis performed at harvest enables the peroneus brevis to compensate for PLT function. Additionally, MRI studies have documented PLT regeneration, which may further underlie the preservation of ankle function following harvest [50].

ACL reconstruction failure is multifactorial, arising from a spectrum of patient-, injury-, and procedure-related factors. Patient anatomic risk factors (e.g., increased posterior tibial slope), concomitant pathology (e.g., subtotal/total meniscal deficiency), technical errors, and premature return to sport are all implicated in the development of ACLR failure [51]. Another major risk factor is the use of an undersized graft. Contemporary practice generally targets a graft diameter of 8 mm or greater to mitigate the risk of failure. Among available autografts, even quadrupled hamstring tendons (HTs) often fall below this threshold [52,53]. To address insufficient diameter, surgeons have adopted multi-strand configurations and fixation systems such as all-inside techniques [54]. The PLT offers a practical solution to the diameter constraint. Owing to its substantial length and caliber, particularly with inframalleolar harvest, the PLT typically achieves the desired diameter with relative ease. Even when tripled, it remains sufficiently long to permit conventional, cortical-suspensory, or interference-based fixation strategies [34]. In our cohort, PLT grafts demonstrated significantly greater diameter than HT grafts. Although graft failure rates did not differ statistically between groups, the PLT appears advantageous in terms of reliably attaining the target graft size.

The HTs play a crucial role in knee biomechanics, as they act as muscles that cross the knee joint, contributing to knee flexion and providing dynamic medial stabilization [55,56]. Consequently, HT harvest may lead to deficits in flexion strength and a shift in the hamstring-to-quadriceps (H/Q) ratio toward quadriceps dominance, a pattern that has been linked to an increased risk of ACL graft failure [57]. Thus, HT harvesting is not entirely innocent with respect to knee function. By contrast, the PLT is anatomically remote from the knee and is unlikely to perturb knee mechanics, which confers a theoretical advantage over HT. In our study, although objective dynamometry was not performed, manual muscle testing suggested a noticeable reduction in knee flexion strength following harvest of the HT compared to PLT. Performance on the single-leg hop test, an overall indicator of lower-limb function, numerically favored the PLT group but did not reach statistical significance. Taken together, these findings suggest that, from a knee function standpoint, PLT may offer certain advantages over HT.

This study has several strengths: a priori sample-size justification; rigorous 1:1 nearest-neighbor matching on age, sex, and follow-up to enhance group comparability; standardized surgical workflows (anteromedial femoral tunnel; suspensory femoral fixation; uniform tibial interference screw; predefined timing of concomitant procedures); and prospectively specified, face-to-face outcome acquisition by the same examiner encompassing knee-specific measures (IKDC, LKS, TAS), stability tests, limb symmetry and strength, as well as ankle-specific function in the PLT cohort (AOFAS, FADI). Nevertheless, important limitations merit consideration. The retrospective, single-center design and surgeon-driven graft selection introduce potential selection and residual confounding biases. Post-match differences in follow-up duration, variability in PLT harvest/tenodesis technique and fixation subtype (fixed vs. adjustable loop), and reliance on manual strength testing rather than isokinetic dynamometry may affect internal validity and sensitivity to detect small effects. Unequal follow-up (HT longer than PLT) may preferentially capture late events in HT, such as re-rupture, and underestimate late donor-site ankle issues in PLT, potentially attenuating or inflating between-group differences. Exclusion of cases that underwent secondary surgery from the comparative functional analysis risks attrition bias; rare events (e.g., re-rupture, deep infection) were likely underpowered. Blinding of assessors was not implemented, imaging biomarkers of graft maturation were not collected, and return-to-sport was individualized rather than protocolized, limiting external applicability. These factors should temper causal inference while the overall pattern, comparable global function with low donor-site ankle morbidity, remains consistent with the matched analyses presented.

## 5. Conclusions

In this matched cohort study of primary ACL reconstructions, PLT autografts achieved clinical and functional outcomes comparable to those of HT autografts. Relative to HT, PLT was associated with a larger graft diameter, better preservation of knee flexion strength on MMT, and lower donor-site ankle morbidity, whereas knee stability, global knee scores (IKDC, LKS), activity level (TAS), overall complications, and re-rupture rates were similar between groups. The higher infection signal observed in the HT group should be interpreted cautiously, given the scarcity of events, imprecision of estimates, and more frequent use of vancomycin graft soaking in the PLT group. Taken together, these results support consideration of PLT as a viable alternative autograft for primary ACL reconstruction. However, given the observational design, potential residual confounding, and the subjectivity of MMT, causal inferences cannot be made. Multicenter prospective trials with standardized rehabilitation, blinded outcome assessment, isokinetic dynamometry, and longer follow-up are warranted to validate these associations and quantify any procedure-specific differences.

## Figures and Tables

**Table 1:** Demographic and clinical characteristics of the patients.

Variables	HT Group (*n* = 82)	PLT Group (*n* = 77)	*p*-Value
Age (years ± SD)	28.6 ± 9.4	27.0 ± 9.4	0.215 ^1^
Sex (*n*, %)			0.506 ^2^
*Male*	64 (77.9%)	61 (79.2%)	
*Female*	18 (21.9%)	16 (20.8%)	
Side (*n*, %)			0.408 ^2^
*Right*	49 (59.8%)	41 (53.2%)	
*Left*	33 (40.2%)	36 (46.8%)	
Weight (kg ± SD)	77.7 ± 15.3	79.0 ± 14.5	0.347 ^1^
Height (cm ± SD)	173.9 ± 8.1	174.6 ± 6.8	0.547 ^3^
BMI (kg/m^2^ ± SD)	25.6 ± 4.6	25.8 ± 4.4	0.323 ^1^
Mechanism of injury (*n*, %)			0.335 ^2^
*Sport injury*	68 (70.7%)	63 (81.8%)	
*Traffic Accident*	9 (11.0%)	7 (9.1%)	
*Industrial injury*	6 (7.3%)	2 (2.6%)	
*Simple Fall*	9 (11.0%)	5 (6.5%)	
ASA Score (*n*, %)			0.650 ^2^
*ASA 1*	45 (54.9%)	45 (58.4%)	
*ASA 2*	37 (45.1%)	32 (41.6%)	
Smoking (*n*, %)			0.347 ^2^
*Active smoker*	38 (46.3%)	30 (39.0%)	
*Quitted/None*	44 (53.7%)	47 (61.0%)	
LOS (days ± SD)	3.4 ± 4.4	1.4 ± 1.0	0.001 ^1^
Follow-up (months ± SD)	27.0 ± 17.7	18.4 ± 15.0	0.001 ^1^

*Abbreviations: BMI: Body Mass Index, ASA: American Society of Anesthesiologists, LOS: Length of Hospital Stay.* ^1^ *Mann–Whitney U test,* ^2^ *Chi-Square Test,* ^3^ *Student T-test*.

**Table 2:** Comparison of Operative characteristics of the patients.

Variables		HT Group (*n* = 82)	PLT Group (*n* = 77)	*p*-Value
Graft diameter (mm ± SD)		8.1 ± 0.7	8.5 ± 0.6	0.004 ^1^
Graft Preparation Technique (*n*, %)				0.001 ^2^
*Double-stranded*		82 (100.0%)	68 (88.3%)	
*Triple-stranded*		0 (0.0%)	9 (11.7%)	
Additional interventions (*n*, %)				
*MM*	*Medial Meniscus intact*	48 (58.5%)	42 (54.5%)	0.490 ^2^
	*Medial Meniscal Repair*	18 (22.0%)	23 (29.9%)	
	*Medial Meniscectomy*	16 (19.5%)	12 (15.6%)	
*LM*	*Lateral Meniscus intact*	66 (80.5%)	59 (76.6%)	0.796 ^2^
	*Lateral Meniscal Repair*	8 (9.8%)	10 (13.0%)	
	*Lateral Meniscectomy*	8 (9.8%)	8 (10.4%)	
Associated MCL Injuries (*n*, %)				0.185 ^2^
*Intact*		70 (85.4%)	72 (93.5%)	
*Grade 1*		7 (8.5%)	4 (5.2%)	
*Grade 2*		5 (6.1%)	1 (1.3%)	
Operation time (min ± SD)		93.1 ± 22.8	93.9 ± 22.0	0.489 ^1^
PLT Harvesting Technique (*n*, %)				NA
*Single inframalleolar*			49 (63.6%)	
*Single supramalleolar*			13 (16.9%)	
*Double incision*			15 (19.5%)	
HT harvesting Technique (*n*, %)				
*Oblique*		82 (100.0%)		
Vancomycin Soaking (*n*, %)				0.001 ^2^
*Yes*		21 (25.6%)	55 (71.5%)	
*No*		61 (74.4%)	22 (28.5%)	

*Abbreviations: MM: Medial Meniscus, LM: Lateral Meniscus, MCL: Medial Collateral Ligament, HT: Hamstring Tendon, PLT: Peroneus Longus Tendon, NA: Not Applicable, SD: Standard Deviation.* ^1^ *Mann–Whitney U Test,* ^2^ *Chi-Square Test.*

**Table 3:** Comparison of functional scores between the groups.

Variables	HT Group (*n* = 82)	PLT Group (*n* = 77)	*p*-Value
Follow-up (months ± SD)	27.0 ± 17.7	18.4 ± 15.0	0.001 ^1^
Preop IKDC (score ± SD)	30.3 ± 16.8	31.9 ± 18.3	0.599 ^1^
Postop IKDC (score ± SD)	76.5 ± 19.8	80.6 ± 18.3	0.185 ^1^
Δ Mean (95% CI)	46.1 (41.7–50.6)	48.6 (43.1–54.2)	
*p*-value (within group)	0.001 ^3^	0.001 ^3^	
Preop LKS (score ± SD)	43.0 ± 20.5	39.8 ± 18.9	0.311 ^2^
Postop LKS (score ± SD)	88.9 ± 12.2	89.5 ± 12.4	0.588 ^1^
Δ Mean (95% CI)	45.9 (41.1–50.6)	49.7 (44.7–54.7)	
*p*-value (within group)	0.001 ^3^	0.001 ^3^	
Preop Tegner Activity Scale	1.8 ± 1.6	2.0 ± 1.7	0.566 ^1^
Postop Tegner Activity Scale	5.4 ± 1.7	5.9 ± 1.7	0.071 ^1^
Δ Mean (95% CI)	3.5 (3.1–4.0)	3.9 (3.4–4.4)	
*p*-value (within group)	0.001 ^3^	0.001 ^3^	
Knee Extension Loss (*n*, %)	0 (0.0%)	1 (1.3%)	0.481 ^4^
Knee Flexion Loss (*n*, %)	56 (68.3%)	7 (9.1%)	0.001 ^4^
Knee Flexion Deficit (°±SD)	9.3 ± 4.0 (*n* = 56)	9.2 ± 1.8 (*n* = 7)	0.806 ^1^
Knee Extension Strength (MMT ± SD)	4.5 ± 0.7	4.7 ± 0.5	0.341 ^1^
Knee Flexion Strength (MMT ± SD)	4.0 ± 0.8	4.7 ± 0.5	0.001 ^1^
Lachman Test (*n*, %)			0.850 ^4^
*Grade 0*	61 (74.4%)	57 (74.0%)	
*Grade 1*	15 (18.3%)	16 (20.8%)	
*Grade 2*	6 (7.3%)	4 (5.2%)	
Anterior Drawer Test (*n*, %)			0.093 ^4^
*Grade 0*	59 (72.0%)	57 (74.0%)	
*Grade 1*	15 (18.3%)	15 (19.5%)	
*Grade 2*	8 (9.8%)	2 (2.6%)	
*Grade 3*	0 (0.0%)	3 (3.9%)	
Δ Thigh circumference (cm ± SD)	1.1 ± 1.5	0.9 ± 1.5	0.255 ^1^
Δ Calf circumference (cm ± SD)	1.4 ± 1.8	1.3 ± 1.6	0.800 ^1^
Tampa Kinesiophobia Scale (score ± SD)	41.9 ± 4.8	40.9 ± 6.1	0.107 ^1^
Tampa Activity Score (score ± SD)	17.4 ± 2.6	17.3 ± 2.8	0.388 ^1^
Tampa Somatic Score (score ± SD)	11.8 ± 1.5	11.4 ± 2.1	0.114 ^1^
Single Leg Hop test (LSI ± SD)	77.4 ± 28.0	82.1 ± 22.1	0.762 ^1^

*Abbreviations: IKDC: International Knee Documentation Committee Subjective Knee Form, LKS: Lysholm Knee Score, MMT: Manual Muscle Testing, LSI: Limb Symmetry Index, SD: Standard Deviation, Δ: Difference,* ^1^ *Mann–Whitney U Test,* ^2^ *Student T Test,* ^3^ *Wilcoxon Signed Rank Test,* ^4^ *Chi-Square Test. Flexion/extension loss = contralateral-referenced deficit ≥ 5° by goniometry.*

**Table 4:** Comparison of donor site morbidity and complications.

Variables	HT Group (*n* = 82)	PLT Group (*n* = 77)	*p*-Value
Ankle Plantar Flexion Loss (*n*, %)	0 (0.0%)	0 (0.0%)	NA
Ankle Dorsal Flexion Loss (*n*, %)	0 (0.0%)	0 (0.0%)	NA
Ankle Inversion Loss (*n*, %)	0 (0.0%)	3 (3.9%)	0.111 ^1^
Ankle Eversion Loss (*n*, %)	0 (0.0%)	0 (0.0%)	NA
AOFAS Ankle-Hind Foot Score (score ± SD)		96.9 ± 7.9	NA
FADI Score (score ± SD)		97.4 ± 8.5	NA
Sural nerve injury (*n*, %)		14 (18.2%)	NA
Saphenous nerve injury (*n*, %)	24 (29.3%)	10 (13.0%)	0.012 ^1^
Postop hemarthrosis aspiration (*n*, %)	12 (14.6%)	10 (13.0)	0.764 ^1^
Re-rupture (*n*, %)	6 (7.3%)	4 (5.2%)	0.747 ^1^
Infection (*n*, %)			0.030 ^1^
None	75 (91.5%)	77 (100.0%)	
Superficial infection	4 (4.9%)	0 (0.0%)	
Deep Infection	3 (3.7%)	0 (0.0%)	

*Abbreviations: AOFAS: American Orthopaedic Foot and Ankle Society, FADI: Foot and Ankle Disability Index, SD: Standard Deviation.* ^1^ *Chi-Square Test.*

**Table 5:** List of previous studies that compared PLT versus HT grafts in primary ACLR.

#	Author & Year	Design	# Patients PLT/HT	Follow-Up (Months)	Functional Outcomes	Secondary Differences
1	Bi et al., 2018 [21]	RCT	62/62	30 m	Similar	Similar
2	Rhatomy et al. 2019 [22]	RCT	24/28	12 m	Similar	Larger graft, less thigh atrophy, no AKP with PLT
3	Vijay et al., 2022 [37]	RCT	23/22	12 m	Similar	Similar
4	Gunadham et al., 2022 [38]	RC	40/40	47 m	IKDC favored HT	AH-PLT grafts were smaller
5	Keyhani et al., 2022 [36]	PC	65/65	24 m	Similar	Larger graft, less thigh atrophy with PLT
6	Agarwal et al., 2023 [39]	PC	96/98	12 m	Similar	Less thigh atrophy with PLT
7	Saeed et al., 2023 [40]	RCT	121/111	24 m	Similar	Earlier return to sport with PLT
8	Gök et al., 2024 [24]	RC	52/54	20 m	Similar	Larger graft, less thigh atrophy with PLT
9	Punnoose et al., 2024 [25]	RCT	25/25	12 m	Similar	Larger graft with PLT
10	Acharya et al., 2024 [35]	RC	30/30	24 m	Similar	Larger graft, shorter graft harvest time with PLT
11	M.Khalid et al., 2024 [41]	RCT	40/40	24 m	NR	Less early pain, better ROM, stronger muscle strength, earlier jogging, and greater satisfaction
12	Ali et al., 2024 [42]	RC	30/30	12 m	Similar	Larger graft with PLT
13	Vyacheslavovich et al., 2024 [43]	PC	55/55	18 m	IKDC & LKS favored HT	Similar
14	Dwidmuthe et al., 2024 [34]	RCT	18/18	6 m	Similar	Longer graft with PLT, less sensory deficit
15	Umer Butt et al., 2024 [23]	RCT	30/30	60 m	Similar	Larger graft with PLT
16	Khalil et al., 2025 [20]	RCT	36/35	6 m	Similar	Larger graft with PLT
17	Current Study, 2025	RC	77/82	22 m	Similar	Larger graft, greater flexion strength, less flexion loss

*Abbreviations: RC: Retrospective Cohort, RCT: Randomized Clinical Trial, PC: Prospective Cohort, PLT: Peroneus Longus Tendon, HT: Hamstring Tendon, NR: Not Reported, AH-PLT: Anterior Half of Peroneus Longus Tendon, ROM: Range of Motion*.

## Data Availability

The datasets are not publicly available. The de-identified data are available upon request to the corresponding author due to privacy, ethical, and legal restrictions protecting patient confidentiality.

## Abbreviations

ACL	Anterior cruciate ligament
ACLR	Anterior cruciate ligament reconstruction
AOFAS	American Orthopaedic Foot & Ankle Society (Ankle–Hindfoot Score)
ASA	American Society of Anesthesiologists
FADI	Foot and Ankle Disability Index
HT	Hamstring tendon
IKDC	International Knee Documentation Committee (Subjective Knee Form)
LSI	Limb Symmetry Index
PLT	Peroneus longus tendon
TAS	Tegner Activity Scale
ROM	Range of motion
